# Long-term cytokine exposure remodels the methylome and transcriptome of recessive dystrophic epidermolysis bullosa keratinocytes – a bioinformatic analysis

**DOI:** 10.3389/fcell.2026.1810599

**Published:** 2026-05-07

**Authors:** Julia I. Hummel, Roland Zauner, Stefanie Gruner, Sonja Dorfer, Michael Ablinger, Vanessa Walch, Caterina Barone, Martin Laimer, Christina Guttmann-Gruber, Josefina Piñón Hofbauer, Ulrich Koller, Johann W. Bauer, Verena Wally

**Affiliations:** 1 Department of Dermatology and Allergology, University Hospital of the Paracelsus Medical University Salzburg, Salzburg, Austria; 2 EB House Austria, Research Program for Molecular Therapy of Genodermatoses, Department of Dermatology and Allergology, University Hospital of the Paracelsus Medical University Salzburg, Salzburg, Austria; 3 Department of Biosciences and Medical Biology, Paris-Lodron University of Salzburg, Salzburg, Austria

**Keywords:** chronic inflammation, DNA methylation, epigenetic reprogramming, keratinocytes, pro-tumorigenic shift, recessive dystrophic epidermolysis bullosa, transcriptome

## Abstract

**Background:**

Recessive dystrophic epidermolysis bullosa (RDEB) is a monogenic skin disorder characterized by severe skin fragility and pronounced clinical variability, even among individuals sharing identical genotypes. Transforming growth factor beta 1 (TGF-β1) and Interleukin-6 (IL-6) signaling have previously been linked to disease severity, but the molecular changes of long-term exposure of patient keratinocytes (KCs) - especially at the level of DNA methylation and gene expression - remain relatively unexplored.

**Methods:**

Data on differential DNA-methylation and gene expression were generated from RDEB-KCs following a 4-week exposure to TGF-β1 or IL-6, as well as after an additional 4-week period without treatment, using the EPIC array and RNA-sequencing, respectively. Cytokine induced epigenetic and transcriptional alterations upon treatment and such that remained stable upon treatment withdrawal were identified using bioinformatic tools based on R/Bioconductor packages for data integration and analysis.

**Results:**

Bioinformatic analysis demonstrated that prolonged cytokine exposure, reflecting chronic inflammation, induced predominantly reversible but also a subset of long-lasting transcriptomic changes in RDEB-KCs. Notably, pathways associated with the RDEB disease phenotype were enriched, with focal adhesion and p53 signaling among the stably altered pathways. Integration of transcriptomic and methylome data identified three genes - *GPR68* and *FBLIM1* modulated by TGF-β1, and *ODF2* responsive to IL-6, as persistently deregulated and demethylated even 4 weeks after treatment termination, a finding that was further confirmed *in vitro*. Moreover, their significant deregulation in RDEB-tumor tissue compared to RDEB-skin controls suggests that cytokine exposure may induce a stable, pro-tumorigenic shift in RDEB-KCs.

**Conclusion:**

Overall, our bioinformatic results highlight stable cytokine-driven molecular alterations in RDEB-KCs that may contribute to disease pathogenesis and potentially revealed candidate pathways and genes for future mechanistic and therapeutic investigation.

## 1 Introduction

Recessive dystrophic epidermolysis bullosa (RDEB) is a rare genodermatosis caused by mutations in the *COL7A1* gene. The resulting partial or complete absence or dysfunction of type VII collagen (C7), a key structural component of anchoring fibrils of the dermal-epidermal junction, leads to a particular fragility of skin and mucous membranes, manifesting in skin blistering, impaired wound healing, and excessive fibrosis. Interestingly, RDEB-patients present with a high disease variability, ranging from rather mild and/or localized RDEB variants to severe RDEB subtypes, for which the development of severe complications such as the fusion of digits (referred to as pseudosyndactyly) and the development of particularly aggressive squamous cell carcinomas (SCC) are common (Has et al., 2020). Even though the type of mutation and the amount of C7 expressed are major determinants of disease severity, this does not always hold true, as there are also patients with identical genetic defects who still exhibit markedly different disease phenotypes (Has et al., 2020; Jarvikallio et al., 1997; Varki et al., 2007; Pathmarajah et al., 2025). Consequently, this suggests that also other factors affect disease evolvement and progression, which are likely to include epigenetic mechanisms. In this context, a pioneering paradigm was the study of monozygotic RDEB twins, who harboured the same genetic mutation and showed similar C7 expression levels, but significantly diverging phenotypes, with one sibling presenting with a severe form of the disease, whereas the other was classified as mildly affected. In the respective study by Odorisio et al., the role of transforming growth factor-beta (TGF-β1) and Interleukin-6 (IL-6) in influencing disease severity was highlighted, by showing increased cytokine signaling in fibroblasts of the more severely affected twin, going along with differential expression of genes linked to the TGF-β1 pathway (Odorisio et al., 2014). Similar results were achieved in a study of RDEB-siblings with identical mutations but diverging phenotypes, conducted by Chacon-Solano et al. (2022). Notably, both cytokines, IL-6 and TGF-β, have repeatedly been associated with epidermolysis bullosa (EB) disease severity. For example, serum IL-6 levels were shown to significantly correlate with EB-severity, evaluated using the Birmingham Epidermolysis Bullosa Severity (BEBS) score (Annicchiarico et al., 2015; Esposito et al., 2016), and the IL-6/IL-10 ratio was shown to be a potential prognostic EB disease marker (Tampoia et al., 2017).

With regard to potential future clinical implications, studies in murine models, using small molecule drugs (losartan (Nystrom et al., 2015)) or biologics (decorin (Cianfarani et al., 2019)) interfering with TGF-β1 signaling, revealed an amelioration of the mice’ phenotype, represented by a delay in digit fusion and reduced skin fibrosis. Furthermore, in a recent clinical trial evaluating the safety and tolerability of losartan over a 10-month treatment period in RDEB children, the treatment resulted in an improvement of clinical scores (*e.g.*, EBDASI (Jain et al., 2017), as well as an improvement of a score indicative for pseudosyndactyly progression, potentially indicating losartan’s fibrosis-modulating mechanism. However, overall, effects were reported to be moderate and variable in clinical improvement (Kiritsi et al., 2024).

In this study, we were interested in molecular mechanisms associated with long-term exposure of skin cells to the pro-inflammatory cytokines TGF-β1 and IL-6, in order to shed light on potentially irreversible effects that might be associated with limited efficacy of cytokine-targeted therapeutic strategies.

Although fibroblasts are the primary drivers of tissue fibrosis and ECM remodeling in RDEB, we focus on the long-term changes induced by TGF-β1 and IL-6 in keratinocytes (KCs). The significance of epithelial cells in this process has recently gained attention; notably, the epithelial-specific deletion of the TGF-β receptor II protects mice from bleomycin-induced pulmonary fibrosis (Li et al., 2011). Moreover, TGF-β1 can stimulate the expression of fibrogenic factors in normal and malignant epithelial cells, with a process associated with pronounced intratumoral fibrosis (Park et al., 2025; Zhang et al., 2019). In addition, epithelial TGF-β1 responses, through effects like upregulation of integrin αvβ6, inhibition of epithelial cell proliferation, and induction of senescence or cell death, likely worsen fibrosis and hinder tissue regeneration (Sheppard, 2015).

TGF-β1 has been well described in the course of studies to be a stimulator of chronicity both *in vitro* and *in vivo* (Liarte et al., 2023; Wang et al., 2006; Katsuno et al., 2019). Here, i.e., a key finding was that long-term exposure of mammary epithelial and cancer cells to TGF-β1 induced stable epithelial-to-mesenchymal transition (EMT) that was not fully reversible upon TGF-β1 withdrawal and the authors raised already the question of a methylation-dependent mechanism underlying this persistence (Katsuno et al., 2019). Indeed, several other studies proved that TGF-β1 lead to long-lasting changes in gene expression, which are in part due to differential methylation events (Negreros et al., 2019; Martin et al., 2014). To date, this aspect, i.e., the long-term cellular effects of TGF-β1, as well as IL-6 in RDEB and the resulting persistent changes on the epigenome and gene expression are relatively unexplored.

To address this gap, in this study, we aimed to elucidate the effects of long-term exposure of RDEB- KCs to TGF-β1 or IL-6 on the transcriptome and methylome, with the goal to gain more insight into pathomechanisms associated with disease severity, and to identify potential biomarkers and drug targets that might mitigate malignant effects of chronic inflammation.

## 2 Materials and methods

### 2.1 Cell culture and cytokine treatments

RDEB-KC cell lines were isolated and cultured from tissues derived from patients (*n* = 3) as described previously (Wimmer et al., 2020; Kocher et al., 2021; Hochmann et al., 2018), upon voluntarily given written, informed consent (Table 1).

**TABLE 1:** List of cell lines used in this study.

Cell line	Experimental group	Age (yrs)	Sex	*COL7A1* mutation	References
RDEB-57-KC	RDEB-KC	3	f	c.427–2A>G/c.4172insC	Wimmer et al. (2020)
RDEB-58-KC	RDEB-KC	0	f	c.2858-2859delAG, homozygous	Sun et al. (2018)
RDEB-223-KC	RDEB-KC	2	f	c.425A>G, homozygous	Kocher et al. (2021)

**FIGURE 1:**
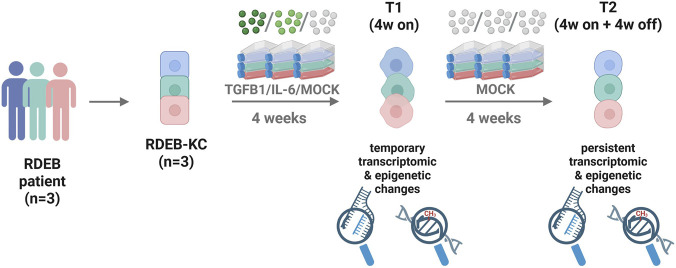
Experimental design for the stimulation of recessive dystrophic epidermolysis bullosa (RDEB)-keratinocytes (KC) with the RDEB-associated inflammatory signals TGF-β1 or IL-6. Subsequent investigation of temporary (T1) and persistently (T2) induced epigenetic changes, assessed through transcriptome and methylome profiling. Illustration created in Biorender®. Wally, V. (2026). https://BioRender.com/qpt7rge.

Ethical approval was granted by the ethics committee of the region of Salzburg (vote number: 415-EP/73/192–2013). Patient KCs were subsequently immortalized by transduction of human papilloma virus proteins E6 and E7 and cultured in defined, serum-free CnT-Prime Epithelial Culture Medium (CELLnTEC, CnT-PR, Bern, Switzerland) at 37 °C with 5% CO_2_ in a humidified incubator and *mycoplasma* tested. For cytokine treatments, cells in T75 flasks were stimulated with either 10 ng/mL TGF-β1 (PeproTech, 100–21) or 10 ng/mL IL-6 (R&D, 7270-IL-025/CF) over a period of 4 weeks (T1). After stimulation termination, the cells were cultivated for additional 4 weeks (T2, Figure 1). Appropriate solvent controls (MOCK) for cytokines were cultured in parallel, and once per week all treatment conditions and respective controls were split simultaneously, with the same number of cells reseeded. Cell pellets for DNA and RNA were collected for both timepoints.

### 2.2 DNA isolation and bisulfite conversion

DNA was isolated using the ReliaPrep™ gDNA Miniprep System (Promega, A5081) following the manufacturer’s instructions. A total of 350 ng of genomic DNA was bisulfite-converted using the EZ DNA Methylation-Gold™ Kit (Zymo Research, D5005) following manufacturer’s recommendations, and the converted DNA was eluted in 10 μL M-Elution Buffer.

### 2.3 DNA methylation profiling and methylome data processing

For the Infinium Methylation EPIC bead chip array (Illumina, 20028878, San Diego, CA, United States), 8 µL of the eluted DNA was loaded and all DNA samples were processed following the Infinium HD Assay Methylation Protocol Guide (15019519 v01). An Illumina NextSeq 550Dx instrument was used for scanning the arrays. Quality control (QC) metrics were generated with Illumina BeadArray Controls Reporter v1.1 Software. QC, data filtering, background correction, and between array quantile normalization were conducted in the statistical software R using the “minfi” Bioconductor package following the recommended workflow outlined by Aryee et al. (2014), Maksimovic et al. (2016). In brief, after QC, a data filter step was applied following recommendations in the Maksimovic workflow. Out of 865,859 probes, 37,575 were dropped based on low detection *p*-value (threshold 1 × 10^−10^), 74,289 were removed based on association with SNPs, cross-hybridization and gender. 753,995 probes passed QC and filtering were considered for further downstream analysis. The R package “limma” (Ritchie et al., 2015) was used to determine differentially methylated CpGs (DMCs). For determination of significant DMCs, a *p*-value <0.01 and |fold-change| ≥ 1.5 threshold was applied. DMCs associated with promoter regions (1st Exon, 5′UTR, TSS1500, and TSS200) were further considered for subsequent integration with differentially expressed genes (DEGs).

### 2.4 RNA isolation and RNA-seq

RNA was extracted using the miRNeasy Tissue/Cells Advanced Mini Kit (Qiagen, 217604, Venlo, Netherlands), according to manufacturer’s recommendations. RNA quality control, polyA enrichment mRNA library preparation (Illumina, NEBnext kit), and sequencing (NovaSeq S1 SR100 flowcell) were outsourced to the Vienna BioCenter.

### 2.5 Semi-quantitative reverse transcription PCR

Semi-quantitative reverse transcription polymerase chain reaction (sqRT-PCR) was performed using GoTaq® qPCR Master Mix (Promega, TM318, Madison, Wisconsin, United States) according to the manufacturer’s protocol and as described elsewhere (Wimmer et al., 2020). RNA was isolated using the miRNeasy Mini Kit (Qiagen), and reverse transcribed using the iScriptTM cDNA Synthesis Kit (BioRad, 1708890, Hercules, California, United States). SqRT-PCR was performed on a BioRad CFX96 instrument. GAPDH was used as reference, and relative expression and significance levels were calculated using the ΔΔCq method. All experiments were performed in technical triplicates.

The following primers were used:

GAPDH fw: 5′ GCC​AAC​GTG​TCA​GTG​GTG​GA 3′, rv: 5′ CAC​CAC​CCT​GTT​GCT​GTA​GCC 3′; GPR68 fw: 5′ GCC​CAG​CTG​TTT​GAG​GTT​TG 3′, rv: 5′ GTC​TGC​AGT​GAT​GTT​CCC​CA 3′; FBLIM1 fw: 5′ AAA​ATC​GAA​TGC​ATG​GGA​AG 3′, rv: 5′ GCA​GGT​TAG​GAA​GGG​AAA​CC 3′; ODF2 fw: 5′ GTG​TCG​CTC​CTG​GTT​TCC​AT 3′, rv: 5′ TTC​ATG​GTT​GGC​TTC​TGG​CA 3′.

### 2.6 Transcriptome data processing

Illumina fastq seq data was aligned employing NextFlow “nf-core rnaseq” pipeline using default parameters to generate the raw count matrix. Analysis of the raw count matrix was conducted in R applying the “edgeR.” The edgeR package was employed to determine which genes have sufficiently large counts to be retained in a statistical analysis. After removing low count filtering (edgeR), 32,818 transcripts were considered for downstream analysis. A principal component analysis (PCA) was conducted using R function *plot.PCA* with top 10,000 genes. Count data were normalized by the method of trimmed mean of M-values (TMM) proposed by Robinson and Oshlack to accommodate for differences in library size using *calcNormFactors* function in edgeR package (Robinson and Oshlack, 2010). Significantly differentially expressed genes/transcripts (DEGs) were determined using a factorial linear model implemented in the limma-voom framework. For nominating DEGs, a cutoff of *p*-value <0.01 and |fold-change| ≥ 1.5 was applied.

For *GPR68*, *FBLIM1*, and *ODF2* expression analysis in RDEB-SCC and RDEB-skin tissue, normalized RNA-seq data generated by Prof. Andy South’s lab (University of Wisconsin-Madison, United States) were retrieved from GEO repository (GEOquery, v2.50.5, GSE111582).

### 2.7 Pathway analysis and enrichment

To analyse the potential biological relevance of changes in gene expression and methylation, top 100 significantly up- and downregulated top genes, as well as DEGs overlapping with DMCs were used (*p* < 0.01 and |fold-change| ≥ 1.5) to query Gene Ontologies (GO, Human Phenotype Ontology) and pathway enrichment (Kyoto Encyclopedia of Genes and Genomes (KEGG), using the Enrichr tool (https://maayanlab.cloud/Enrichr/) (Xie et al., 2021).

In addition, enrichment of significant DEGs for individual gene sets associated to pathways with specific relevance to RDEB-pathology were explored based on DEGs filtered by *p* < 0.01 and |fold-change| ≥ 1.5. The following gene set collections were used: Focal adhesion (KEGG 2021 Human); Skin fibrosis (Elsevier Pathway Collection); Syndactyly MP:0000564 (MGI Mammalian Phenotype Level 42024), Cutaneous Syndactyly HP:0012725 (Human Phenotype Ontology), Impaired/Delayed/Abnormal Wound Healing MP:0001792/MP:0002908/MP:0005023 (MGI Mammalian Phenotype Level 42024), Burn Wound Healing WP5055 (WikiPathways 2024 Human), Regulation of/Wound Healing, Spreading of Epidermal Cells GO:0035313/GO:1903689 (GO Biological Process 2025); Epithelial to Mesenchymal Transition in Cancer: Overview (Elsevier Pathway Collection); p53 signaling pathway (KEGG 2021 Human/BioPlanet 2019); Squamous cell carcinoma HP:0002860 (Human Phenotype Ontology), Squamous cell carcinoma of skin (DisGeNET).

## 3 Results

### 3.1 Long-term exposure of RDEB-KCs to TGF-β1 and IL-6 induced global transcriptomic changes

**FIGURE 2:**
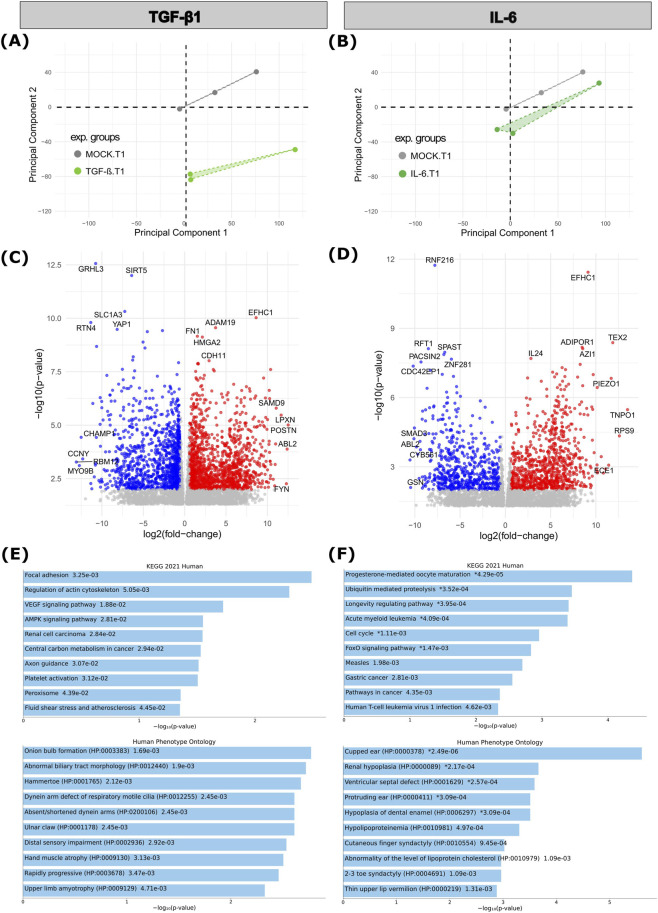
Genome-wide transcriptome analysis of RDEB-KC treated 4 weeks with TGF-β1 or IL-6. **(A,B)** Principal Component Analysis (PCA) based on RNA-seq data for **(A)** TGF-β1 and **(B)** IL-6 treated RDEB-KC, respectively. **(C,D)** Volcano plot showing differentially expressed genes (DEGs) in RDEB-KCs treated with **(C)** TGF-β1 or **(D)** IL-6 for 4 weeks, with downregulated genes highlighted in blue and upregulated genes in red (*p* < 0.01; |fold-change| ≥ 1.5). The ten top up- and downregulated genes following treatment are labeled. **(E,F)** Gene Ontology (GO, Human Phenotype Ontology) and pathway enrichment analysis (Kyoto Encyclopedia of Genes and Genomes (KEGG)) of top 100 DEGs in long-term **(E)** TGF-β1 or **(F)** IL-6 stimulated RDEB-KCs (*p* < 0.01; |fold-change| ≥ 1.5).

To investigate the impact of long-term exposure of RDEB-KCs to TGF-β1 and IL-6 stimulation on gene expression, we incubated KCs for 4 weeks in the presence or absence of the respective cytokine and performed RNA-seq (Figure 1). PCA of resulting RNA-seq data showed a clear separation of stimuli versus MOCK-treated KCs in dimension 2 (PC2, Figures 2A,B). Next, genes expressed differentially in treated KCs over control were extracted. For TGF-β1, we identified 1,415 significantly upregulated genes and 1,211 downregulated genes upon treatment (Supplementary Table S1; *p* < 0.01; |fold-change| ≥ 1.5). Of note, in the TGF-β1 group, among the most significantly upregulated genes we found well known TGF-β1 target genes, like “periostin” (*POSTN*), a potent ECM organizer that has previously been described to be increased in RDEB-fibroblasts and also in the circulation of RDEB patients (Chacon-Solano et al., 2019), “A Disintegrin And Metalloproteinase Domain 19” (*ADAM19*), a trans-membrane protein with shedding activity that has frequently been seen overexpressed in fibrotic diseases promoting fibroblast activation (Meng et al., 2024), and “Fibronectin 1” (*FN1*), a primary component of fibrotic tissue (Griggs et al., 2017). Among the most significantly downregulated genes prominent candidates were “Grainyhead Like Transcription Factor 3” (*GRHL3*), a protein playing important roles in skin homeostasis and wound healing (Gordon et al., 2014), and “Sirtuin 5” (*SIRT5*), a protein being attributed a protective role against fibrosis (Wang et al., 2023; Zullo et al., 2021) (Figure 2C).

For IL-6, 864 genes were significantly up-, and 606 downregulated (Supplementary Table S2; *p* < 0.01; |fold-change| ≥ 1.5). Among the top upregulated genes were relevant players like *IL-24*, a well-known pro-inflammatory cytokine that modulates epithelial and immune cell responses, e.g., being highly expressed in chronic wounds (Bosanquet et al., 2012; Mitamura et al., 2020), as well as “Piezo Type Mechanosensitive Ion Channel Component 1” (*PIEZO1*), a mechanosensor in KCs relevant in inflammation, wound healing, scarring and fibrosis (Zhu et al., 2023; Xue et al., 2025). Top IL-6 downregulated genes included “SMAD Family Member 3” (*SMAD3*), a protein that has been found to have context-dependent tumor suppressive and promotive functions in SCCs and a key regulatory role in IL-6/STAT3 driven fibrosis in systemic sclerosis (Han and Wang, 2011; O'Reilly et al., 2014), “gelsolin” (*GSN*), an actin-binding protein with anti-inflammatory functions, also known to promote wound healing (Wittmann et al., 2018; Witke et al., 1995), and “Laminin Subunit Beta 3” (*LAMB3*), a component of the laminin-332 trimer, which plays a key role in wound healing and which is known to cause junctional EB if mutated (Has et al., 2020; Michopoulou et al., 2020) (Figure 2D).

Gene Ontology (GO)-term enrichment analysis revealed that top 100 differentially expressed genes (DEGs, *p* < 0.01 and |fold-change| ≥ 1.5) upon TGF-β1 treatment significantly affected biological processes like “focal adhesion”, “regulation of actin cytoskeleton” or “VEGF signaling pathway” (Figure 2E). EnrichR analysis of IL-6 induced DEGs highlighted pathways like “ubiquitin mediated proteolysis”, “longevity regulation pathway” or “cell cycle”, as well as ontologies like “cutaneous finger syndactyly”, “2–3 toe syndactyly” or “hypolipoproteinemia” (Figure 2F).

**FIGURE 3:**
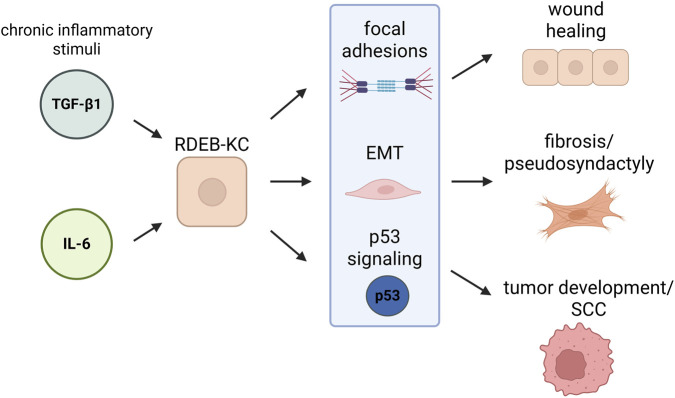
Cytokine stimulation of RDEB-KC affects signaling pathways involved in RDEB pathogenesis. Illustration created in Biorender®. Wally, V. (2026). https://BioRender.com/je28wxf. RDEB recessive-dystrophic epidermolysis bullosa, KC keratinocytes, EMT epithelial-to-mesenchymal transition, SCC squamous cell carcinoma.

Further exploration of pathways relevant to RDEB-pathology (*e.g.*, regulation of focal adhesion, fibrosis, syndactyly, wound healing, EMT, p53 signaling, and tumor development and/or progression; Figure 3) revealed several of the associated genes to be differentially expressed (*p* < 0.01; |fold-change| ≥ 1.5) after 4 weeks of treatment (Table 2).

**TABLE 2:** Overview of RDEB-relevant pathways and associated differentially expressed genes (DEGs; *p* < 0.01; |fold-change| ≥ 1.5) at T1. Number (n) of genes overlapping (o) with total (t) pathway gene list is given as total numbers (n (o/t)) and percentage (%)). Bold and underlined: Genes that remained stably deregulated upon cytokine withdrawal (T2). DEG differentially expressed genes; EMT epithelial-to-mesenchymal transition.

Pathway	DEGs (T1: TGF-β1)		n (o/t) %	DEGs (T1: IL-6)		n (o/t) %
	Down	UP		Down	UP	
Focal Adhesion	ACTG1, ACTN4, CAV1, CCND1, CCND2, FLNB, LAMA3, MET, MYL12B, **MYLK**, PAK1, PAK4, PPP1R12A, PPP1R12B, VAV3	ACTN1, COL1A1, COL1A2, COL4A1, COL4A2, COL6A2, COL6A3, CTNNB1, FLNB, **FLNC**, FN1, FYN, **ILK**, ITGA11, ITGA2, ITGA5, ITGAV, ITGB3, **ITGB5, ITGB6**, LAMB3, LAMC2, MAP2K1, PARVB, PDGFC, PDGFRA, **PIK3CD**, PIK3R1, PIP5K1A, PRKCA, PXN, RAC2, SRC, THBS1, **THBS3**, VEGFA, VEGFC, XIAP	**52/201 25.9%**	ACTG1, **CCND1**, FLNB, LAMB3, MAPK8, PIK3CD, **PIK3R1**, PTK2, VCL	AKT1, AKT3, CTNNB1, ERBB2, **FLNB**, FYN, ITGB3, **ITGB5**, LAMB3, PIK3CD, PIK3R1, THBS3, VEGFA, VEGFC, ZYX	**20/201 10.0%**
Fibrosis	EDN1, FOS, IL6R, MAP2K4, MAP3K14	EDNRA, IL6, MAP2K1, MMP1, MMP2, MMP9, PDGFC, PDGFRA, STAT3, TGFB1, TGFBR2, TRAF2, VEGFA	**18/67 26.9%**	MAP2K4, MAPK8, SMAD3	EDNRA, MMP3, TRAF2, VEGFA	**7/67 10.4%**
Syndactyly	ACTG1, CRIM1, **GLI3**, GRHL2, IRF6, KRT14, NXN, PVRL4	ASPH, CCBE1, COL1A1, DCHS1, DLG5, GREM1, LBR, PORCN, SNAI2, TCF7, WNT5A	**19/113 16.8%**	ACTG1, APBB2, DKK1, FGFR2, KCTD1, LMBR1, WDR11	CCBE1, FBN2, KCTD1, LBR, **RBM10**, WDR19	**12/113 10.6%**
Wound Healing	CAV1, CLASP2, DST, FGFR3, HSPB1, IL6R, KLF4, KRT15, LEPR, LGALS7, PHLDB2, PRKCH, S100A9, SDC4, SKP2, SLPI, **TGFB2**, TP53	AEBP1, CD44, COL1A1, COL1A2, COL5A1, DYSF, FBN1, FERMT1, FN1, **GSN**, IL6, INHBA, ITGA5, ITGAV, ITGB3, **ITGB5**, MMP2, MMP9, RAC2, SDC2, SNAI2, TGFBI, VEGFA	**42/176 23.9%**	CDK16, FGFR2, GIPC1, GSN, HSPB1, PLEC, SCEL, SMAD3	AEBP1, AKT1, CD44, DST, **DYSF**, **GSN**, ICAM1, ITGB3, **ITGB5**, MMP3, SKP2, VEGFA	**19/176 10.8%**
EMT	CDH1, CLDN1, CRB3, DDR1, DLL1, DSP, IL6R, IRS1, NOTCH3, OCLN	AXL, CDH2, FN1, FOXC2, GLI2, IL6, JAG1, MMP2, MMP9, NOTCH4, PDGFRA, PIK3R1, SERPINE1, SNAI2, SRC, STAT3, VIM	**27/90** **30.0%**	OCLN, PTK2, PTK2B, VCL	AKT1, ERBB2, HIF1A, **PIK3R1**	**8/90 8.9%**
p53 Signaling	AIFM2, ARID3A, BCL6, BDKRB2, BTG2, CAV1, CCND1, CCND2, CTSD, DGCR8, DUSP1, EDN2, HSPA1B, IGFBP3, JMY, MET, NFYC, PERP, PRDM1, RNF144B, SERPINB5, TP53, TP73, VDR	BCL2L1, CCNE1, **MAP4K4**, **MDM2**, MDM4, MMP2, NFYC, PRKAB1, RRM2B, SERPINE1, SHISA5, SNAI2, **STEAP3**, **TFDP1**, THBS1, **TP53I3**, VCAN, ZNF385A	**42/179** **23.5%**	ARID3A, **CCND1, CD82**, DKK1, DROSHA, IRF5, MAP4K4, VDR	BBC3, BCL2L1, **CD82, MAP4K4**, MDM4, PML, PRKAB1, SHISA5, SMARCA4, **STEAP3, TFDP1, ZNF385A**	**18/179 10.1%**
Squamous Cell Carcinoma	AQP3, CCND1, GRHL3, KLF4, KRT16, KRT17, MYC, NR3C1, OCLN, **PIK3CD**, RIPK4, RPS6, S100A8, SERPINB4, SPRR1A, TP53, TP73, TRIM16, TSC1, WNT10A, YAP1	CARD11, CD44, DAP, EPHB2, ERCC1, FAP, FYN, **IL24**, IL6, LIMK1, MAP3K9, MMP1, NR3C1, PIK3CD, PLAT, SEC16A, SRC, ST3GAL1, STAT3, TGFB1, TGFBR2, THBS1, **TMC6**, VEGFA, VEGFC, VIM, XIAP, XPC	**47/214 22.0%**	**CCND1**, GRHL3, LGR6, NR3C1, OCLN, PIK3CD, SPRR1A, **STK19**, TRIM16, WNT10A	CARD11, CD44, DEF8, ERBB2, FAP, FHL1, FYN, **IL24**, LIMK1, MAP3K9, **NR3C1**, PIK3CD, PLAT, SEC16A, SERPINB4, TINF2, TSC1, VEGFA, VEGFC, **YAP1**	**28/214** **13.1%**

Bold: Genes that remained stably deregulated upon cytokine withdrawal (T2).

### 3.2 Differentially expressed genes that remain stable beyond treatment

**FIGURE 4:**
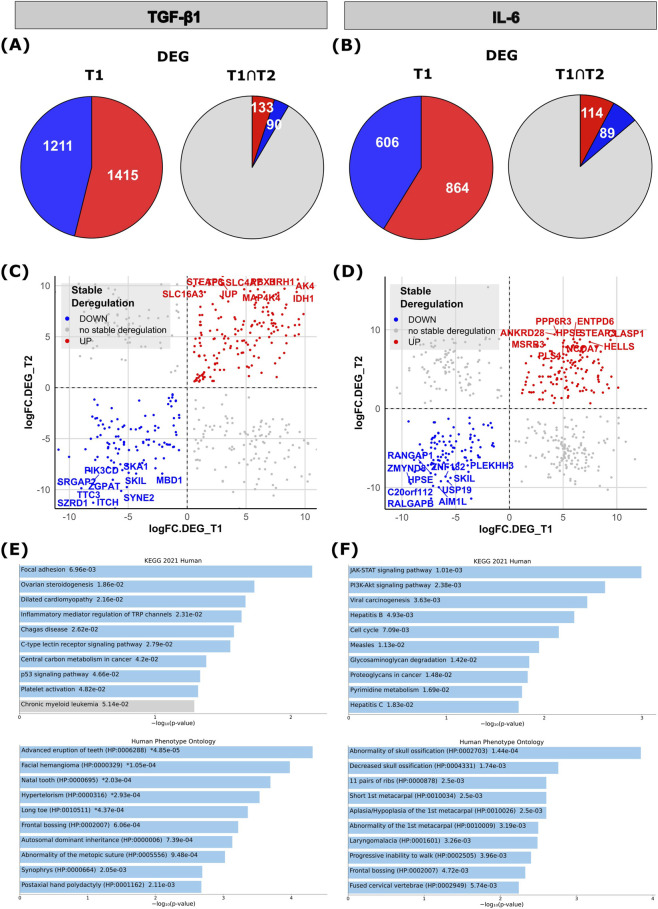
Analysis of differentially expressed genes (DEGs) in TGF-β1 or IL-6 stimulated RDEB-KC at T2. **(A,B)** Left pie charts represent all DEGs detected at T1, (upregulated (red): **(A)** TGF-β1: *n* = 1,415; **(B)** IL-6: *n* = 864; downregulated (blue): **(A)** TGF-β1: *n* = 1,211; **(B)** IL-6: *n* = 606) genes. Right pie charts illustrate those genes that remained stably deregulated at T2 (upregulated (red): **(A)** TGF-β1: *n* = 133; **(B)** IL-6: *n* = 114; downregulated (blue): **(A)** TGF-β1: *n* = 90; **(B)** IL-6: *n* = 89; *p* < 0.01; |fold-change| ≥ 1.5). **(C,D)** Scatter plot illustrating **(C)** TGF-β1 induced and **(D)** IL-6 induced DEGs, consistently downregulated (blue dots) or upregulated (red dots) at T1 and T2 (*p* < 0.01; |fold-change| ≥ 1.5). The top 10 DEGs with the highest ± fold change at T2 are labeled. **(E,F)** Gene Ontology (GO, Human Phenotype Ontology) and pathway enrichment analysis (Kyoto Encyclopedia of Genes and Genomes (KEGG)) of DEGs that remained stable upon termination of **(E)** TGF-β1 or **(F)** IL-6 stimulation in RDEB-KCs (*p* < 0.01; |fold-change| ≥ 1.5). Colored bars correspond to terms with significant *p*-values (<0.05).

It has previously been shown that long-term TGF-β1 exposure may induce transcriptomic changes that are irreversible, even upon withdrawal of TGF-β1 (Katsuno et al., 2019). In order to explore this phenomenon also in the context of RDEB, we analyzed changes in gene expression that persisted even 4 weeks upon treatment termination (T2). Of the 2,626 DEGs upon TGF-β1 treatment, RNA-seq revealed 220 genes to remain significantly deregulated (Supplementary Table S3), including 133 upregulated and 90 downregulated genes (Figure 4A). Of the 1,470 DEGs upon IL-6 treatment, 193 genes remained to be differentially expressed (Supplementary Table S4), of which 114 were upregulated and 89 downregulated (Figure 4B; *p* < 0.01; |fold-change| ≥ 1.5).

Interestingly, among the persistently deregulated genes we found genes whose functions suggest potential relevance to RDEB-associated complications (*e.g*., inflammation, wound healing, tumor development), like the “Solute Carrier Family 16 Member 3” (*SLC16A3*), a monocarboxylate transporter, supporting glycolytic metabolism and associated with poor prognosis in multiple cancers (Sun et al., 2020), or “Mitogen-Activated Protein Kinase Kinase Kinase Kinase 4” (*MAP4K4*), a kinase involved in systemic inflammation, focal adhesion dynamics and cancer (Singh et al., 2021; Yue et al., 2014). Prominent candidates of TGF-β1-induced persistently downregulated genes were “Itchy E3 Ubiquitin Protein Ligase” (*ITCH*), a regulator of epidermal keratinocyte differentiation that was also shown to prevent chronic skin inflammation (Rossi et al., 2006; Theivanthiran et al., 2015), and the “SKI Like Proto-Oncogene” (*SKIL*), a negative regulator of the TGF-β1/SMAD pathway (Tecalco-Cruz et al., 2012) (Figure 4C).

Among the genes that remained upregulated despite IL-6 withdrawal, we found “Heparanase” (*HPSE*), an enzyme that degrades heparan sulfate in the extracellular matrix (ECM) and basement membrane, thereby having important functions in tissue remodeling, wound healing and inflammatory signaling (Mayfosh et al., 2021), and “Helicase, Lymphoid Specific” (*HELLS*), a chromatin remodeling protein that interacts with DNA methyltransferases, which was also shown to regulate epidermal homeostasis (Wang et al., 2020). On the other hand, “Ral GTPase Activating Protein Non-Catalytic Subunit Beta” (*RALGAPB*) involved in oncogenic Ral signaling (Rasche et al., 2025), or “Absent In Melanoma 1 Like” (*AIM1L*), an actin-binding protein and suppressor of epithelial cell motility and invasion (Haffner et al., 2017) continued to show strong downregulation (Figure 4D).

To explore the similarities in how each cytokine influences gene expression, we examined the overlap of genes differentially regulated by both TGF-β1 and IL-6, identifying those that are affected by both (Supplementary Figure S1; Supplementary Tables S14, S15).

GO-term enrichment analysis of DEGs that were stably deregulated also after TGF-β1 withdrawal highlighted pathways like “focal adhesion”, “C-type lectin receptor signaling pathway” or “p53 signaling pathway”, and ontologies such as “facial hemangioma” or “postaxial hand polydactyly” (Figure 4E). When investigating DEGs that remained stably deregulated upon IL-6 withdrawal, pathways like the “JAK-STAT signaling pathway”, the “PI3K-Akt signaling pathway”, or “proteoglycans in cancer”, as well as ontologies such as “laryngomalacia”, or “progressive inability to walk” were enriched (Figure 4F).

Furthermore, several of the DEGs at T2 were also associated with the previously analyzed RDEB-relevant pathways (stable DEGs are highlighted in Table 2). It is noteworthy that among all DEGs, genes associated with HDAC1/2/3 signaling were deregulated at both T1 and T2, suggesting a sustained regulatory shift (Supplementary Table S5). As HDAC signaling plays a key role in epigenetic remodeling and chromatin organization, this motivated us to further investigate DNA-methylation dynamics and integrate DNA-methylation with gene-expression data.

### 3.3 Effect of keratinocyte exposure to TGF-β1 and IL-6 on the methylome

**FIGURE 5:**
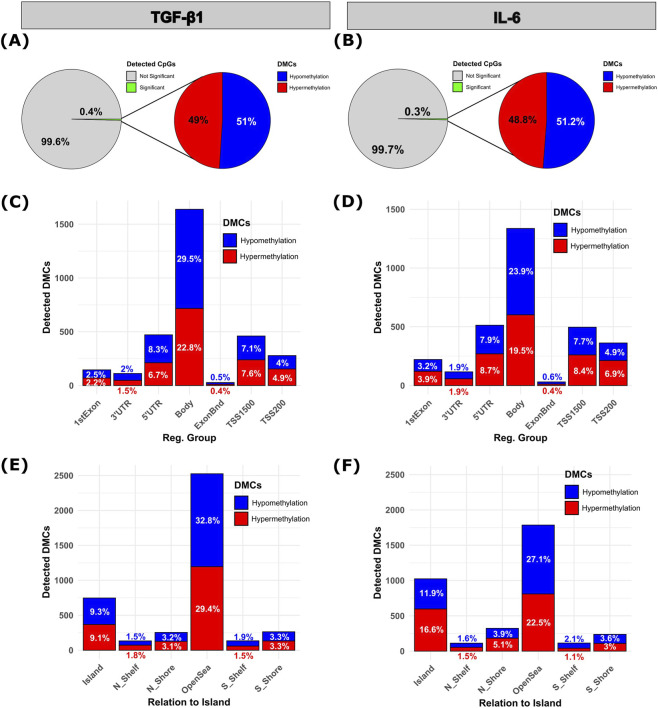
Distribution analysis of differentially methylated CpGs. **(A,B)** Analysis of all detected CpGs reveals that 0.4% in **(A)** TGF-β1 stimulated, and 0.3% in **(B)** IL-6 stimulated RDEB-KC, exhibit significant differential methylation. Among differentially methylated CpGs, 49% were hypermethylated (red) and 51% were hypomethylated (blue) in **(A)** TGF-β1 stimulated RDEB-KC, while 48.8% were hypermethylated and 51.2% were hypomethylated in **(B)** IL-6 stimulated RDEB-KC. **(C–F)** Bar plots showing the distribution of detected DMCs among the different genomic locations, as well as their proximity to CpG island. Bars are color-coded, with red indicating the hypermethylated and blue indicating the hypomethylated CpG fraction. Numbers represent the percentage of detected DMCs in each segment relative to the total number of detected DMCs.

We were interested in TGF-β1 and IL-6 induced differential DNA-methylation in RDEB-KC after 4 weeks of respective cytokine treatments. Out of all detected CpG probes, 0.4% showed a significant differential methylation upon TGF-β1 stimulation, of which 49% were hypermethylated (*n* = 1,548) and 51% hypomethylated CpGs (*n* = 1,613) (Figure 5A; Supplementary Table S6). In comparison, IL-6 treatment resulted in 0.3% of differentially methylated CpGs, with 48.8% showing a hypermethylation (*n* = 1,233) and 51.2% hypomethylation (*n* = 1,294) (Figure 5B; Supplementary Table S7; *p* < 0.01; |fold-change| ≥ 1.5). Of those, 4.7% (TGF-β1) and 7.1% (IL-6) were located within the 1^st^ exon, 15% (TGF- β1) and 16.6% (IL-6) within the 5′UTR, 14.7% (TGF-β) and 16.1% (IL-6) within TSS1500, and 8.9% (TGF-β1) and 11.8% (IL-6) within TSS200. The majority of detected DMCs, with 52.3% for TGF-β1 and 43.4% for IL-6, were located within gene body (Figures 5C,D). On the other hand, regarding their distribution relative to CpG island proximity, we observed that a substantial proportion of methylation changes (TGF-β1: 18.4%, IL-6: 28.5%) induced by TGF-β1 or IL-6 were located within CpG islands in addition to such found in open sea regions (TGF-β1: 62.2%, IL-6: 49.6%) (Figures 5E,F). Overall, methylation analyses indicated that the DMCs identified in TGF-β1- and IL-6-treated RDEB-KCs showed a comparable distribution across genomic regions and their relation to CpG island.

### 3.4 Integrated analysis of DNA methylation and gene expression

**FIGURE 6:**
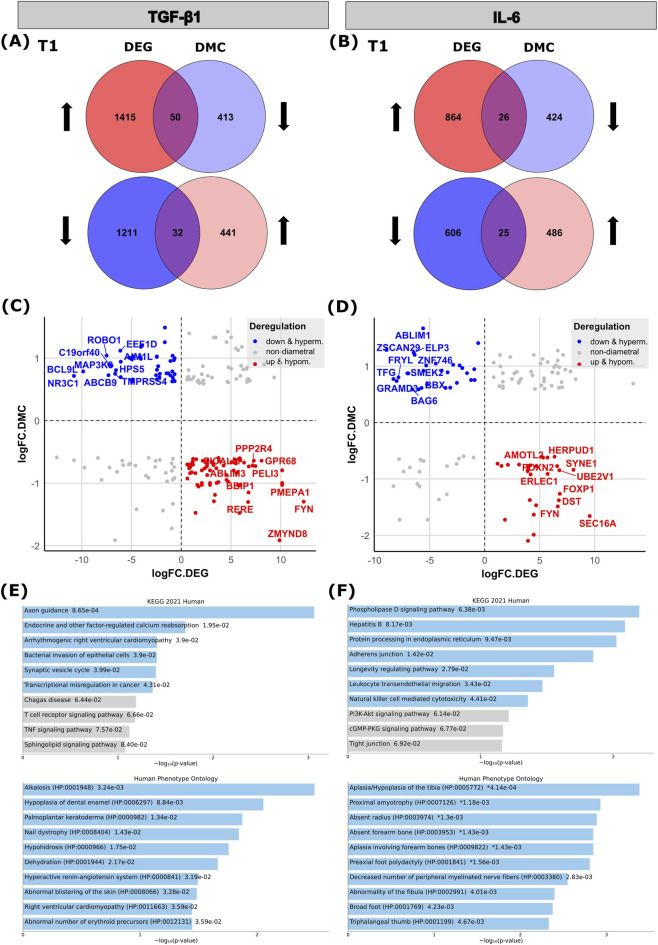
Integrated analysis of gene expression and DNA methylation of RDEB-KC treated 4 weeks with TGF-β1 or IL-6. **(A,B)** Venn diagrams showing diametral intersect between differentially expressed genes (DEGs) and genes with differentially methylated CpGs (DMCs) at T1. **(A)** 133 genes show significant differential expression (DE) together with significant differential methylation in TGF-β1 treated RDEB-KC cells, of which 50 are upregulated/hypomethylated and 32 are downregulated/hypermethylated. **(B)** For IL-6 treated RDEB-KC cells, 97 genes overlap between significant DEGs and significant DMCs, of which 26 are upregulated/hypomethylated and 25 are downregulated/hypermethylated (*p* < 0.01; |fold-change| ≥ 1.5). **(C,D)** Scatter plot representing overlapping DE-DMC genes for **(C)** TGF-β1 and **(D)** IL-6, with downregulated/hypermethylated genes shown in blue, upregulated/hypomethylated in red, and non-diametral genes in grey (*p* < 0.01; |fold-change| ≥ 1.5). The top 10 up-/downregulated genes of the intersect are labeled. **(E,F)** Gene Ontology (GO, Human Phenotype Ontology) and pathway enrichment analysis (Kyoto Encyclopedia of Genes and Genomes (KEGG)) of diametral overlapping DEG-DMC genes following **(E)** TGF-β1 or **(F)** IL-6 stimulation in RDEB-KC (*p* < 0.01; |fold-change| ≥ 1.5). Colored bars correspond to terms with significant *p*-values (<0.05).

To elucidate the functional impact of epigenetic regulation, we performed an integrated analysis of DNA methylation and gene expression. Based on the known principle that promoter hypermethylation can restrict transcription factor binding and suppress gene expression, while hypomethylation tends to promote gene activation (Jones, 2012), we focused on inverse (diametral) correlations between methylation changes and gene expression. For this purpose, we restricted our analysis on promoter-associated DMCs (1^st^ Exon, 5′UTR, TSS1500, and TSS200), yielding 854 DMCs in response to TGF-β1 stimulation (Supplementary Table S8) and 910 DMCs in response to IL-6 (Supplementary Table S9). Among the genes upregulated in TGF-β1-stimulated RDEB-KC cells (T1), 50 exhibited corresponding hypomethylated DMCs, while 32 downregulated genes were associated with hypermethylated DMCs (Figure 6A; Supplementary Table S10). Stimulation with IL-6 resulted in 26 upregulated genes associated with hypomethylated DMCs, and 25 downregulated genes linked to hypermethylation (Figure 6B; Supplementary Table S11).

Representatives of the top TGF-β1-induced downregulated/hypermethylated genes (Figure 6C) were “B-Cell CLL/Lymphoma 9-Like Protein” (*BCL9L*), a β-catenin co-activator relevant in tumor progression by regulating WNT- and TGF-β1-signaling (Vafaizadeh et al., 2021) and “Roundabout Guidance Receptor 1” (*ROBO1*), which is together with SLIT proteins relevant in cell-cell and cell-matrix interactions, as well as tumor progression (Christofidou et al., 2024). Among the upregulated/hypomethylated genes relevant examples are “FYN Proto-Oncogene” (*FYN*), a tyrosine kinase that regulates cell adhesion and cytoskeletal dynamics, modulating TGF-β1-driven epithelial responses linked to EMT and fibrosis, including DNA damage related signaling (Veith et al., 2021; Yu et al., 2020), “G Protein-Coupled Receptor 68” (*GPR68*), a pH-sensing receptor previously been linked to skin cancers, inflammation and epithelial barrier function (Klatt et al., 2020; De Valliere et al., 2015; De Valliere et al., 2022), and “Prostate transmembrane protein androgen induced 1” (*PMEPA1*), a well-known TGF-β1 response mediator involved in skin fibrosis, a promoter of EMT and associated with poor survival in several cancers (Wardhani et al., 2021; Watanabe et al., 2019). Moreover, in a study comparing transcriptomes of RDEB-wounds versus non-wounded RDEB-skin, *PMEPA1* has also been found to be deregulated (Onoufriadis et al., 2022).

Following IL-6 stimulation, among the top downregulated/hypermethylated genes were “BCL2 Associated Athanogene 6” (*BAG6*), a chaperone-like protein involved in tumor progression by regulating extracellular vesicle biogenesis (Alashkar Alhamwe et al., 2024), or “Actin Binding LIM Protein 1” (*ABLIM1*), an actin binding protein playing a role in cytoskeletal organization that has also been shown to be downregulated in EB-simplex (Liovic et al., 2009). Prominent candidates of the most upregulated/hypomethylated ones were “SEC16A Homolog A” (*SEC16A*), a scaffold protein required for endoplasmic reticulum export that has been genetically associated with cutaneous SCC risk (Chahal et al., 2016), “Forkhead Box P1” (*FOXP1*), a transcription factor known to get induced by IL-6/STAT3 signaling and having important roles in fibrosis, senescence, wound healing and inflammation (Yang et al., 2025; Hu et al., 2022), and again “*FYN*” (Figure 6D, ranked by logFC of DEGs).

GO-term enrichment analysis of diametral DEG/DMC genes upon TGF-β1 stimulation revealed enriched pathways like “Bacterial invasion of epithelial cells”, or “Transcriptional misregulation in cancer”. Additionally, of specific interest were enriched ontologies like “nail dystrophy”, “palmoplantar keratoderma” or “abnormal blistering of the skin” (Figure 6E). Diametral DEG/DMCs following IL-6 stimulation resulted in an enrichment of pathways like “phospholipase D signaling”, “Leukocyte transendothelial migration” or “adherens junctions”, and ontologies such as “preaxial foot polydactyly”, or “decreased number of peripheral myelinated nerve fibers” (Figure 6F).

### 3.5 Stable gene deregulation and methylation in response to TGF-β1 and IL-6

**FIGURE 7:**
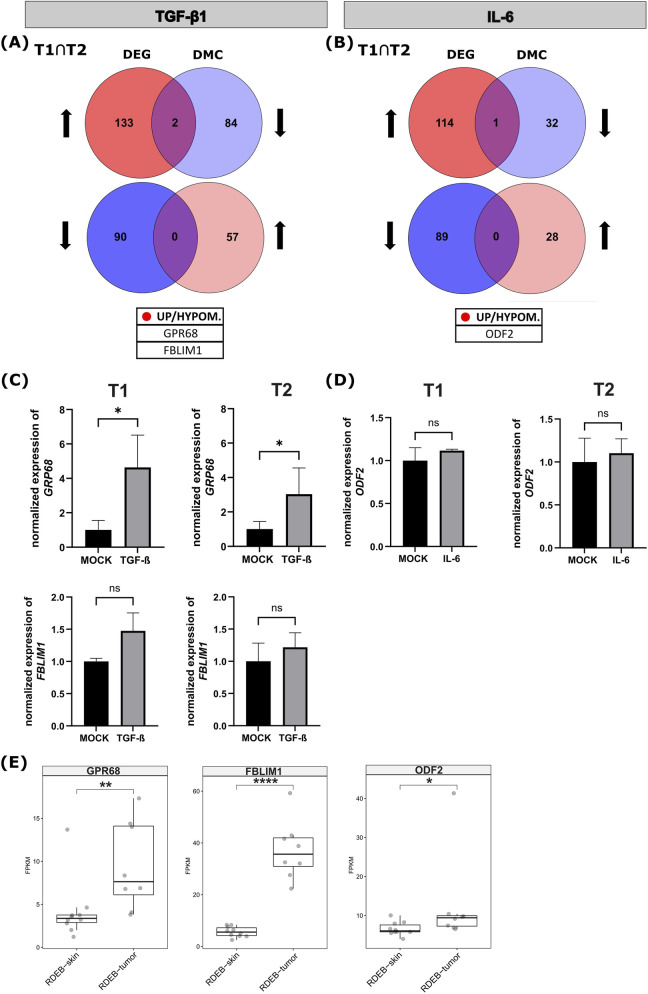
Integrated gene expression and DNA-methylation analysis of RDEB-KCs treated with TGF-β1 or IL-6 reveals stable changes post-stimulation and suggests a pro-tumorigenic switch. **(A,B)** Venn diagrams illustrating diametral overlap between differentially expressed genes (DEGs) and genes with differentially methylated CpGs (DMCs) that remained consistently deregulated and differentially methylated 4 weeks after withdrawal of cytokine stimulation (T2). Two genes overlapped between significantly upregulated DEGs and significantly hypomethylated DMCs upon **(A)** TGF-β1 withdrawal, namely, *GPR68* and *FBLIM1*, whereas no genes remained stably downregulated and hypermethylated at T2. Following **(B)** IL-6 withdrawal, *ODF2* remained significantly upregulated and hypomethylated (*p* < 0.01; |fold-change| ≥ 1.5). **(C,D)** Validation of gene expression by semi-quantitative reverse transcription polymerase chain reaction (sqRT-PCR) confirmed **(C)** TGF-β1 induced upregulation of *GPR68*, which was significant at T1 and remained significantly elevated at T2, even after 4 weeks of TGF-β1 withdrawal. FBLIM1 expression was also increased at both timepoints, although not significantly. Similarly, **(D)** IL-6 treatment induced sustained upregulation of *ODF2* at T1 and T2. Bars represent mean expression values derived from *n* = 3 biological replicates. Error bars represent standard error of mean (SEM), paired t-test on *GAPDH* normalized expression values. **(E)** Transcriptome analysis generated from bulk RNA-seq by **(A)**. South lab (Cho et al., 2018) revealed significantly increased levels of FPKM normalized *GPR68*, *FBLIM1* and *ODF2* expression levels in RDEB-SCC tumors compared to normal RDEB skin. Statistical significance was determined using non-parametric Wilcox test. Each dot represents tissue obtained from RDEB patients. *p < 0.05, **p < 0.01, ****p < 0.0001, ns non-significant, GPR68 G Protein-Coupled Receptor 68, FBLIM1 Filamin Binding LIM Protein 1, ODF2 Outer Dense Fiber Of Sperm Tails 2, FPKM Fragments Per Kilobase per Million mapped fragments.

We next investigated the diametric overlaps between differentially expressed, and differentially methylated genes at T2. Among the 133 TGF-β1 induced stably upregulated genes, two genes–*GPR68* and “Filamin Binding LIM Protein 1” (*FBLIM1*), an adhesion-associated cytoskeletal protein, also mentioned in the context of the EB Kindler-syndrome (Has et al., 2009) – also showed a persistent hypomethylation in T2. In contrast, there was no gene that showed an overlap between downregulated and hypermethylated genes 4 weeks after TGF-β1 withdrawal (Figure 7A; Supplementary Table S12). Among the 114 genes that remained upregulated following IL-6 withdrawal, only one gene also showed persistent hypomethylation at T2, namely, “Outer Dense Fiber Of Sperm Tails 2” (*ODF2*), whose function in the skin is only sparsely described in the literature. Again, none of the 89 downregulated genes, displayed sustained hypermethylation following IL-6 withdrawal (Figure 7B; Supplementary Table S13). Notably, when comparing the expression of the three consistently deregulated/demethylated genes, along with selected top TGF-β1 and IL-6 induced targets, between primary healthy control (HC) KCs and primary RDEB-KCs, we observed no significant differences, except for SIRT1 (Supplementary Figure S2). To further validate the persistence of the cytokine-induced gene expression changes, we performed sqRT-PCR on the three overlap-genes at both timepoints, T1, representing 4 weeks of cytokine stimulation and T2, after an additional 4-week withdrawal period. Upon TGF-β1 treatment, *GPR68* expression was significantly upregulated at T1 and remained significantly elevated at T2, indicating a sustained deregulation even after cytokine removal. *FBLIM1* and the IL-6 induced *ODF2* showed a similar trend of increased mRNA levels at both timepoints, which, however, did not reach significance (Figures 7C,D). These results suggest that prolonged exposure to TGF-β1 and IL-6 can lead to stable gene expression changes in RDEB-KCs. Interestingly, reanalyzing available tissue data provided by Cho et al. (2018) revealed significant deregulation of our selected overlap genes in RDEB tumor tissue compared to normal RDEB skin. Consistent with our RDEB-KC cytokine stimulation data, *GPR68*, *FBLIM1*, and *ODF2* were significantly elevated in RDEB tumor tissue compared to normal RDEB skin (Figure 7E). Under consideration of their roles in other cancers, they represent potential candidates for future investigations.

Overall, our study highlighted that long-term exposure of RDEB-KCs to TGF-β1 or IL-6 induces stable changes in gene expression and DNA methylation. Notably, these persistently differentially expressed and methylated genes are also deregulated in RDEB tumors compared to normal RDEB skin, suggesting a stable phenotypic switch of RDEB-KCs toward a pro-tumorigenic state.

## 4 Discussion

Chronic inflammation is a key complication in RDEB-patients, shaping RDEB-expressivity significantly. Consequently, several treatment approaches have emerged that target RDEB-associated inflammation in general, or pro-inflammatory cytokines, including TGF-β1 and IL-6 individually (Oleogel-S10, Decorin, diacerein, losartan, unrestricted somatic stem cells (USSC), angiotensin inhibitor, RTA408, or tocilizumab) (Schwieger-Briel et al., 2019; Cianfarani et al., 2019; Dorfer et al., 2025; Nystrom et al., 2015; Anderson-Crannage et al., 2025; Joseph et al., 2025; Cohen-Nowak et al., 2022; Naruse et al., 2024). Still, to date, only three drugs have reached market approval, among them one, Filsuvez® (Oleogel-S10), which targets inflammation in the context of wound healing (Schwieger-Briel et al., 2019). Notably, across inflammatory skin diseases, clinical trials of anti-inflammatory agents have frequently reported moderate or variable efficacy, which might, among others, reflect disease/patient heterogeneity, the complexity of inflammatory signaling cascades and molecular determinants (Tsoi et al., 2022; Lax et al., 2024; Teng et al., 2023; Kiritsi et al., 2024). Also, epigenetic genome alterations like changes in DNA methylation patterns, histone modifications, non-coding RNA’s, and chromatin remodeling activity, may alter the expression of therapy-relevant genes and thereby influence treatment efficacy (Sadida et al., 2024; Singh et al., 2022; Daskalaki et al., 2025).

TGF-β1 and IL-6 are cytokines that play key roles in, e.g., wound healing, tissue fibrosis, shaping of immune responses, and in promoting cell proliferation and differentiation (Deng et al., 2024; Grebenciucova and VanHaerents, 2023). An imbalance or dysregulation of these cytokines has been associated with various diseases, among them cardiovascular, skeletal, connective, autoimmune and metabolic disorders (Massague and Sheppard, 2023; Grebenciucova and VanHaerents, 2023). In the skin, dysregulation contributes to chronic inflammation, poor wound healing outcomes, fibrosis, and ultimately to the development of SCCs (Liarte et al., 2020). While IL-6 is a potent pro-inflammatory signal, TGF-β1 seems to have a context dependent dual role and can have both, anti-inflammatory and tumor suppressive, but also pro-inflammatory and tumor promoting functions (Massague and Sheppard, 2023). In the context of RDEB, IL-6 and TGF-β1 have been associated with disease severity and the occurrence of complications, such as fibrosis, wound chronification, and the development of SCCs (Akasaka et al., 2021; Tampoia et al., 2017; De Gregorio et al., 2023; Condorelli et al., 2019; Illmer et al., 2023). In order to gain more understanding of the impact of permanent inflammatory stimulation of KCs, we exposed RDEB-KCs to TGF-β1 or IL-6 for several weeks, and explored changes in gene expression signatures with a focus on such genes, for which also events of differential methylation could be observed that potentially conferred persistent deregulation also upon withdrawal of the inflammatory stimuli. Importantly, to exclude a possible pre-imprintment of cells, we used KCs derived from very young RDEB-patients (mean age 1.6 years, Table 1), as these were not pre-exposed to years of inflammation.

When comparing the effects of the two cytokines, TGF-β1 induced a stronger transcriptional response than IL-6 in RDEB-KCs, as shown in the PCAs and total numbers of DEGs at T1 (Figure 2; Supplementary Tables S1, S2). A comparable number of DEGs remained deregulated after withdrawal of the respective cytokine (T2), and both cytokines induced an enrichment of pathways relevant to RDEB. However, TGF-β1 exposure predominantly enriched pathways related to cytoskeletal organization, cell adhesion, EMT and cancer, consistent with its established roles in fibrosis and tumor progression. In contrast, IL-6 primarily promoted pathways associated with inflammation, infection responses, and stress, which is consistent with its role as a pro-inflammatory signaling molecule. Notably, the identified enriched signaling pathways also included those linked to syn- and polydactyly, a relevant finding as pseudosyndactyly is a primary complication in RDEB. In addition, also other RDEB-relevant ontologies were enriched, including “abnormal blistering of the skin”, “nail dystrophy” or “palmar hyperkeratosis”, and also ontologies relating to skeletal and limb abnormalities, underlining the potential role of our experimental cytokines in disease expressivity and progression.

Already evident after 4 weeks of treatment, “focal adhesion” was among the most prominently enriched pathways, with associated DEGs remaining even after treatment withdrawal. Given the role of focal adhesions (FA) in connecting cells to the ECM, this points towards a relevance of FA dysregulation in RDEB wound healing via their impact on cell migration, adhesion and mechanical sensing (Pora et al., 2019; Katoh, 2024). Also, the “p53 signaling” pathway remained enriched upon cytokine withdrawal suggesting a persistent cellular stress response, which in a chronic setting may increase selective pressure for genomic instability and the risk of cancer development (Abuetabh et al., 2022; Gudkov and Komarova, 2016). This goes in line with findings of Lee et al., who found upregulation of the p53 signaling pathway in an RDEB-patient with recurrent cutaneous SCC (Lee et al., 2024). In RDEB patients, KCs are continuously exposed to a complex inflammatory and fibrotic microenvironment, where multiple cytokines, including TGF-β1 and IL-6, act simultaneously (Anderson-Crannage et al., 2023). Although our study examined each cytokine individually, there is a certain overlap in genes responding to the respective cytokines. Genes commonly induced by both cytokines may represent core disease-defining pathways (Supplementary Figure S1; Supplementary Tables S14, S15), while a subset of genes remained persistently deregulated across treatments. Among the persistently deregulated genes are such that have been associated with pathological processes such as impaired wound healing, fibrosis, and altered epithelial-to-mesenchymal signaling. However, future studies are necessary to investigate the pathomechanliistic relevance of our findings, as well as the impact of additional factors, such as the loss of C7 and paracrine signals from fibroblasts and immune cells.

To shed further light on epigenetic mechanisms associated with TGF-β1 and IL-6 treatments, we screened for DEGs with corresponding diametral methylation events in regions with most relevance to regulation of gene expression. *GPR68* and *FBLIM1*induced by TGF-β1, and *ODF2*, responsive to IL-6 were identified as genes that stayed both differentially methylated and differentially expressed even after prolonged culturing in the absence of additional cytokine exposure. The pH-sensing receptor GPR68, which is activated by acidic environments, is a recognized regulator of fibrosis in fibroblasts and has also been implicated in promoting EMT and inflammation in epithelial cells (Nagel et al., 2022). However, its specific function within the context of RDEB has not yet been explored. Similarly, the role of ODF2 in the physiology of the skin remains largely unexamined.

FBLIM1 is important for cell adhesion and maintaining cytoskeletal stability and has been previously identified as a binding partner of kindlin-1 in the context of Kindler syndrome. Despite this association, FBLIM1 expression is not dependent on kindlin-1, and FBLIM1 knockout mice exhibit a normal phenotype with intact skin (Moik et al., 2011; Lai-Cheong et al., 2008). More recent research has revealed a novel function for FBLIM1 as a key regulator of autophagy, a process through which it may also promote cancer progression (Cai et al., 2024). Whether our observations are specific to RDEB or reflect a general response to cytokine treatments remains to be determined by performing parallel experiments in healthy KCs.

Overall, this study provides a data-driven look at the methylome and transcriptome response of *in vitro*-stimulated KCs, with a particular focus on long-term effects of RDEB-KC exposure to pro-inflammatory cytokines TGF-β1 and IL-6. Bioinformatic data analysis revealed that distinct pathways appeared to be altered long-term, even in the absence of any further cytokine stimuli. Integrating our transcriptome and methylome data highlighted distinct genes to be permanently deregulated, which we could confirm *in vitro*. Notably, these biomarkers also showed a significantly altered expression in RDEB-tumor tissue compared to RDEB-skin, potentially suggesting a stable pro-tumorigenic switch of the RDEB-KCs upon cytokine exposure.

In conclusion, with this primarily bioinformatic analysis, we provide novel insights into the impact of long-term exposure of RDEB-KC to cytokines that have previously been described to correlate with RDEB disease severity. Further studies are necessary to determine the relevance of our findings on a potential drug candidate’s mechanism of action, and to assess whether and how long-term deregulation of specific genes may impact treatment outcomes.

## Data Availability

Raw transcriptome and genome-wide methylome sequencing data are not available to protect patient privacy; anonymized data will be made available upon reasonable request. The authors declare that all other data are contained within the manuscript and Supplementary Materials.

## Glossary

ABLIM1: Actin Binding LIM Protein 1
ADAM19: A Disintegrin And Metalloproteinase Domain 19
AIM1L: Absent In Melanoma 1 Like
BAG6: BCL2 Associated Athanogene 6
BCL9L: B-Cell CLL/Lymphoma 9 Like
C7: Type VII Collagen
DEG: Differentially Expressed Gene(s)
DMC: Differentially Methylated CpG(s)
EB: Epidermolysis Bullosa
ECM: Extracellular Matrix
EMT: Epithelial-to-Mesenchymal Transition
FBLIM1: Filamin Binding LIM Protein 1
FN1: Fibronectin 1
FOXP1: Forkhead Box P1
FYN: FYN Proto-Oncogene
GO: Gene Ontology
GPR68: G Protein-Coupled Receptor 68
GRHL3: Grainyhead Like Transcription Factor 3
GSN: Gelsolin
HELLS: Helicase, Lymphoid Specific
HPSE: Heparanase
IL-6: Interleukin-6
ITCH: Itchy E3 Ubiquitin Protein Ligase
KC: Keratinocyte
KEGG: Kyoto Encyclopedia of Genes and Genomes
LAMB3: Laminin Subunit Beta 3
MAP4K4: Mitogen-Activated Protein Kinase Kinase Kinase Kinase 4
ODF2: Outer Dense Fiber of Sperm Tails 2
PCA: Principal Component Analysis
PIEZO1: Piezo Type Mechanosensitive Ion Channel Component 1
PMEPA1: Prostate Transmembrane Protein, Androgen Induced 1
POSTN: Periostin
QC: Quality control
RALGAPB: Ral GTPase Activating Protein Beta Subunit
RDEB: Recessive Dystrophic Epidermolysis Bullosa
ROBO1: Roundabout Guidance Receptor 1
SCC: Squamous Cell Carcinoma
SEC16A: SEC16 Homolog A
SIRT5: Sirtuin 5
SKIL: SKI Like Proto-Oncogene
SLC16A3: Solute Carrier Family 16 Member 3
SMAD3: SMAD Family Member 3
sqRT-PCR: Semi-quantitative Reverse Transcription Polymerase Chain Reaction
TGF-β1: Transforming Growth Factor Beta 1
USSC: Unrestricted Somatic Stem Cell
