# Supplementary material for: Long-term cytokine exposure remodels the methylome and transcriptome of recessive dystrophic epidermolysis bullosa keratinocytes – a bioinformatic analysis
Source: Front Cell Dev Biol. 2026 May 7;14:1810599. doi: 10.3389/fcell.2026.1810599 (PMC13189922; doi:10.3389/fcell.2026.1810599)
Supplement: Supplementary file 1 [file Supplementaryfile1.docx]

Supplementary Material

# Supplementary Figures and Tables

## Supplementary Figures


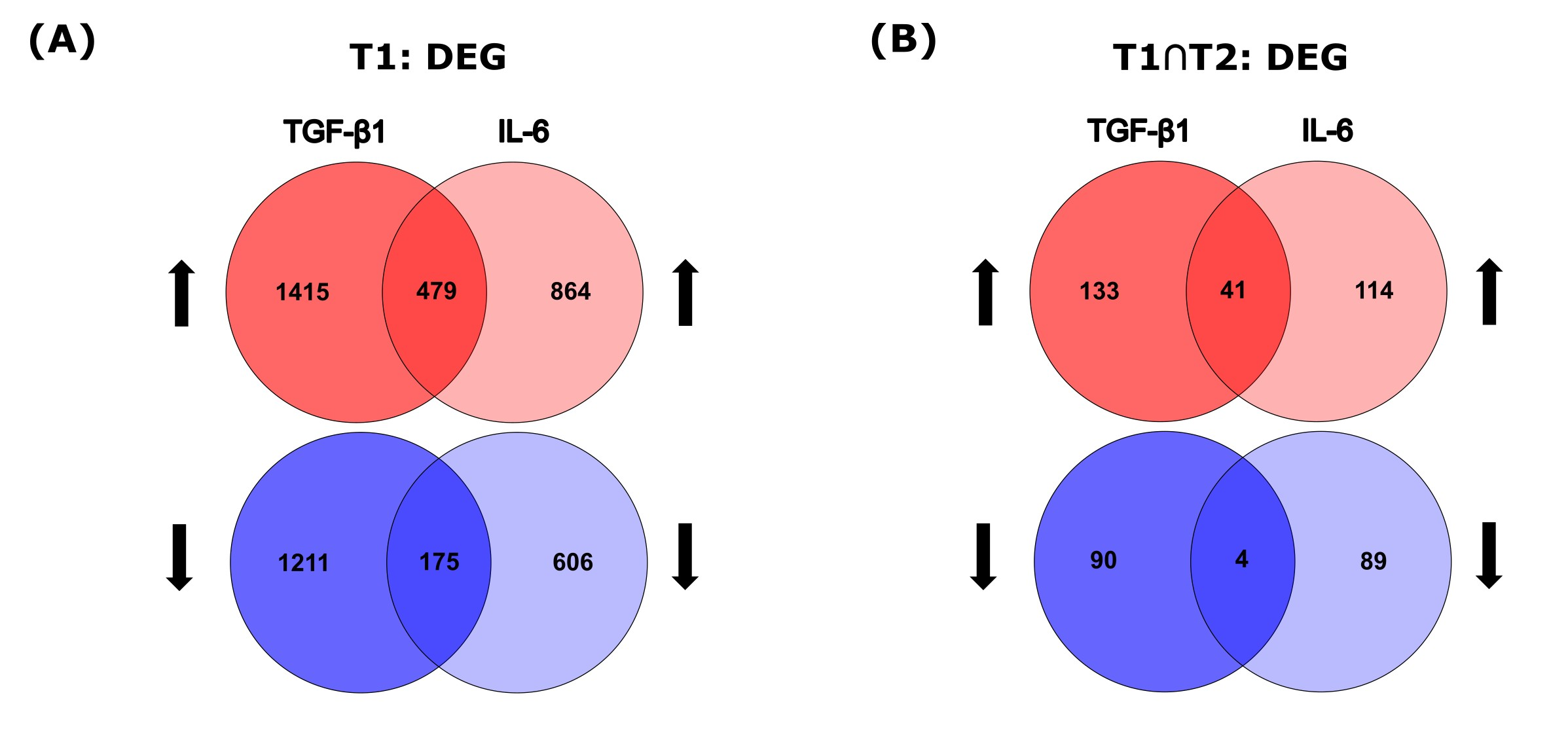


**Supplementary Figure 1.** Overlap of differentially expressed genes (DEGs) induced by TGF-β1 as well as IL-6 in RDEB-KCs. (A) Venn diagrams showing 479 upregulated genes that are induced by both TGF-β1 as well as IL-6 (red), and 175 downregulated overlap genes at T1 (blue). (B) Venn diagrams showing 41 stably upregulated genes upon TGF-β1 as well as IL-6 withdrawal (red), and 4 persistently downregulated overlap genes (blue) (*p* < 0.01; |fold-change| ≥ 1.5).


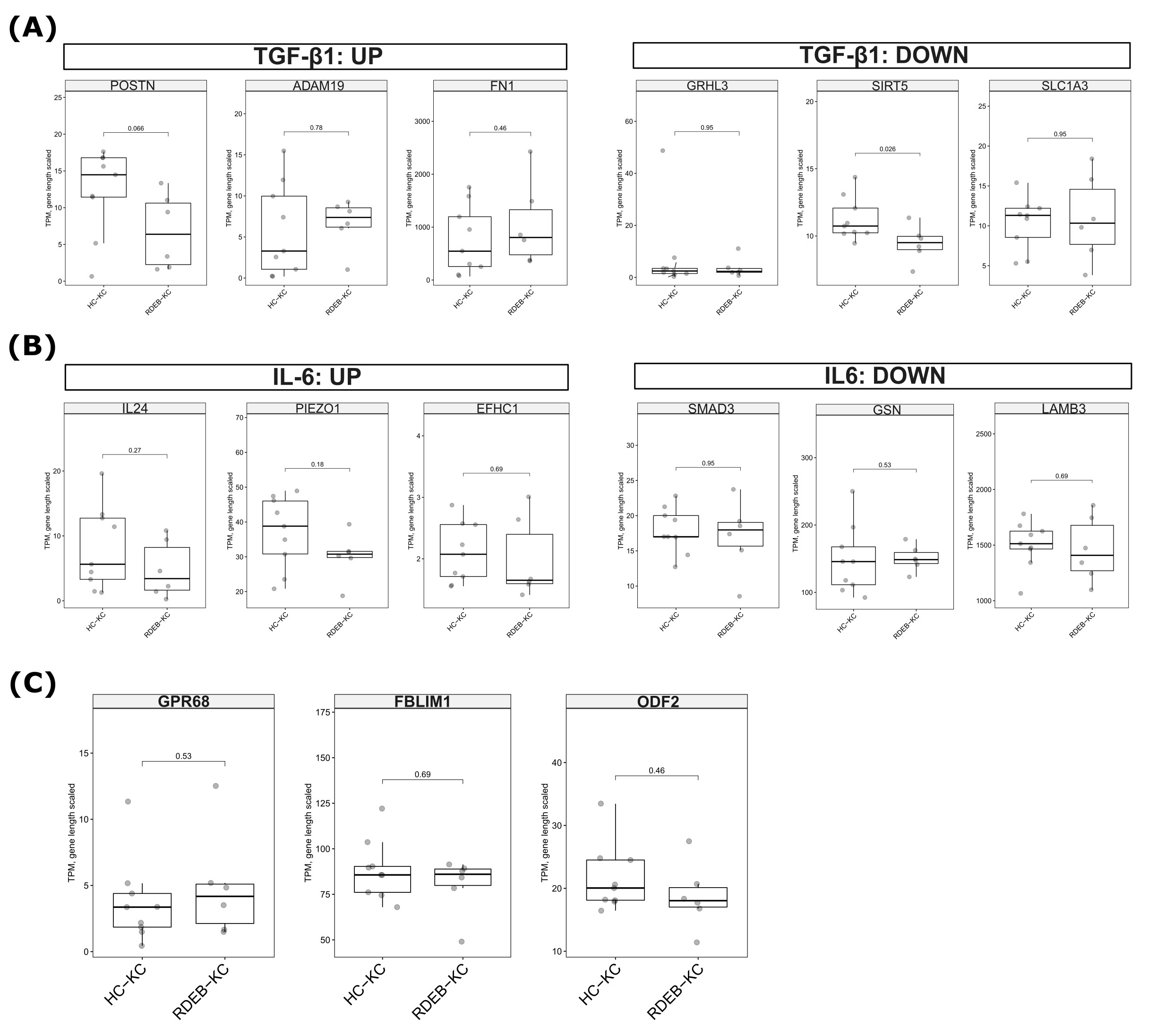


**Supplementary Figure 2.** Bulk-RNA-seq results of selected targets in primary healthy control (HC) versus RDEB-KCs. (A-B) Baseline expression of the top three up- and downregulated genes upon TGF-β1 (A) and IL-6 (B) treatment. (C) Expression levels of the three persistently deregulated/demethylated genes, *GPR68*, *FBLIM1*, and *ODF2* in HC compared to RDEB-KCs. Statistical significance was determined using non-parametric Wilcox test. Each dot represents an individual primary cell sample from a different donor.
